# Epithelioid hemangioendothelioma of the spine: an analysis of imaging findings

**DOI:** 10.1186/s13244-022-01197-5

**Published:** 2022-03-26

**Authors:** Yongye Chen, Xiaoying Xing, Enlong Zhang, Jiahui Zhang, Huishu Yuan, Ning Lang

**Affiliations:** 1 Department of Radiology, Peking University Third Hospital, 49 North Garden Road, Haidian District, Beijing, 100191 People’s Republic of China; 2 Department of Radiology, Peking University International Hospital, 1 Life Science Park, Life Road, Haidian District, Beijing, 102206 People’s Republic of China

**Keywords:** Epithelioid hemangioendothelioma, Spine, Magnetic resonance imaging, Tomography

## Abstract

**Background:**

Epithelioid hemangioendothelioma (EHE) is a low-grade malignant vascular neoplasm with the potential to metastasize. Primary EHE of the spine is very rare and an accurate diagnosis is crucial to treatment planning. We aim to investigate the imaging and clinical data of spinal EHE to improve the understanding of the disease.

**Methods:**

We retrospectively analyzed the imaging manifestations and clinical data of 12 cases with pathologically confirmed spinal EHE. The imaging features analyzed included number, locations, size, border, density, signal, majority of the lesions, expansile osteolysis, residual bone trabeculae, sclerotic rim, vertebral compression, enhancement.

**Results:**

Patients included 5 female and 7 male patients (mean age: 43.0 ± 19.6 years; range 15–73 years). Multiple lesions were noted in 1 case and single lesion was noted in 11 cases. The lesions were located in the thoracic, cervical, lumbar, and sacral vertebrae in 7, 3, 1, and 1 cases, respectively. They were centered in the vertebral body and posterior elements in 9 and 3 cases, respectively. Residual bone trabeculae, no sclerotic margin, and surrounding soft-tissue mass were noted in 11 cases, each, and mild expansile osteolysis and vertebral compression were noted in 10 and 6 cases, respectively. MRI was performed for 11 patients, all of whom showed isointensity on T1WI, hyperintensity or slight hyperintensity on T2WI, and hyperintensity on fat-suppressed T2WI. A marked enhancement pattern was noted in 10 cases.

**Conclusion:**

Spinal EHE tend to develop in the thoracic vertebrae. EHE should be considered when residual bone trabeculae can be seen in the bone destruction area, accompanied by pathological compression fracture, no sclerotic rim, and high signal intensity for a vascular tumor on T2WI.

## Key points

- The spinal epithelioid hemangioendotheliomas (EHE) are extremely rare.
- Diagnosis of spinal EHE is crucial to the treatment.
- Some imaging features may valuable for the diagnosis of spinal EHE.

## Background

Epithelioid hemangioendothelioma (EHE), which originates from vascular endothelial or pre-endothelial cells, is a low-grade malignant neoplasm with the potential to metastasize. The behavioral and histopathologic features of EHE have been reported to rank between those of hemangiomas and angiosarcomas [1]. EHE manifest as epithelioid endothelial cells arranged in nests or cords with infiltrative growth into surrounding tissues. Immunohistochemically, EHE cells usually express endothelial markers, such as EGR, CD31, CD34, and F VIII–Rag [2, 3].

EHE can develop in any part of the body, and most commonly develop in parenchymatous organs such as the liver and lung, and also in bone and soft tissue [4]. Primary EHE of the spine is very rare and clinically it mainly manifests as local pain and neurological symptoms caused by compression of the spinal cord or nerve root. Treatment options for spinal EHE include preoperative embolization, surgical resection, radiotherapy, and chemotherapy. Accurate diagnosis is of great significance in treatment planning. For example, preoperative embolization is recommended because spinal EHE is a very vascular tumor which can be associated with significant intraoperative bleeding. Preoperative embolization can shrink the tumor and reduce the probability of intraoperative hemorrhage, which can afford a clearer visualization of the surgical field and increase the success rate of complete tumor resection [5, 6]. The diagnosis of spinal EHE mainly depends on histopathology. However, patients would benefit if preoperative imaging can provide some valuable information that hints towards EHE.

On account of the low morbidity rates associated with spinal EHE, only few case reports [5–10] and case series [11–14] have been reported in the literature. To the best of our knowledge, no series of imaging manifestations of spinal EHE is available in the literature. In this study, we retrospectively reviewed the imaging manifestations of 12 patients with EHE of the spine, to provide some valuable information for the imaging diagnosis and improve the in-depth comprehension on it.

## Methods

### Subjects

This retrospective study was approved by the institutional review board of our hospital, and written informed consent was waived.

A retrospective analysis of the case data of patients with spinal EHE from January 2008 to November 2018 was performed. The inclusion criteria were as follows: (1) diagnosis of spinal EHE by pathological biopsy; (2) CT and/or MRI examinations were performed before treatment. The exclusion criteria were as follows: (1) the lesion area had been subject to any treatment including surgical resection, radiotherapy, etc. before CT or MRI examinations; (2) poor image quality that could not be analyzed.

### Image acquisition

CT scans were obtained using Discovery 64-slice VCT (GE Medical System) or Somatom Definition Flash dual-source CT (Siemens). Scanning parameters were as follows: tube voltage, 120 kV; tube current, 163–300 mA; section thickness, 3 mm; and spiral pitch, 0.980. MRI was performed using Discovery MR750 3.0 T (GE Healthcare) or Magnetom Trio 3.0 T (Siemens) at a section thickness of 3 mm. The imaging protocol included axial T2WI, coronal T2WI, sagittal T2WI, sagittal T1WI, and sagittal fat-suppressed T2WI. The imaging parameters were as follows: T1WI repetition time (TR) = 400–800 ms, echo time (TE) = 10–30 ms; and T2WI TR = 2500–4000 ms, TE = 50–120 ms. The contrast agent, 0.2 [ml/kg] Gd-DTPA, was injected through the elbow vein at a rate of 1 ml/s by using a power injector. After injection, axial T1WI fat-suppressed scanning was performed, and the parameters were as follows: TR = 571–652 ms and TE = 9.8–11.2 ms.

### Image analysis

Image analysis was performed by two musculoskeletal radiologists with more than 10 years of experience. The factors analyzed included the number of lesion (single/multiple), locations, size, border, density (compared with muscle), signal (compared with the spinal cord signal), majority of the lesions (vertebral body/posterior elements), expansile osteolysis, residual bone trabeculae, sclerotic rim, vertebral compression, pattern of enhancement. Discrepancies were resolved by a consensus between the two radiologists.

## Results

### Patients

The patient population included 5 female and 7 male patients aged 15 to 73 years (mean age was 43.0 ± 19.6 years). The clinical information of patients is shown in Table 1. All 12 patients underwent CT examination before treatment. Eleven patients underwent MR examination before treatment, and 10 patients underwent contrast-enhanced MR scanning.

**Table 1:** Summarized data for all the cases in our study

No	Age (years)/Sex	Location	Diameter (cm)	Number of lesions	Symptoms/duration	Management	FU (M)/outcome
1	42/M	T2	6	Solitary	Back pain/6 months; Bilateral lower-extremity numbness/10 days	WD	15/NED
2	21/F	T9	6.1	Solitary	Back pain/5 months; Bilateral lower-extremity numbness and weakness/1 months	WD + RT	22/NED
3	44/M	C2	4.4	Solitary	Neck stiffness/3 years	WD + RT	48/NED
4	37/M	C4	3.2	Multiple	Fore-chest pain/1 year; Neck pain and movement limitation/4 months	WD	7/NED
5	47/M	T6	4	Solitary	Lower back pain/9 months; Bilateral lower-extremity numbness/9 months	WD	85/NED
6	16/F	L4-5	7	Solitary	Lumbocrural pain/3 years	WD	44/NED
7	72/F	T3-4	3.9	Solitary	Lower back pain/1 year; Bilateral lower-extremity numbness and weakness/10 days	RT	29/Partial regression
8	15/M	T3	3.6	Solitary	Back pain/6 months	WD	Loss to FU
9	51/F	S1-2	7.3	Solitary	Sacrococcygeal pain/2 years; Right plantar numbness/2 years	WD	Loss to FU
10	30/M	C2-3	6.5	Solitary	Neck pain/1 year	preoperative RT + WD	Loss to FU
11	73/M	T10	2.9	Solitary	Back pain/3 years	WD	26/NED
12	67/F	T5	3.9	Solitary	Chest pain//9 months	WD	40/NED

EHE: epithelioid hemangioendothelioma; M: male; F: female; C: cervical; T: thoracic; L: lumbar; S: sacra; RT: radiation therapy; WS: wide surgery; FU: follow-up; NED: no evidence of disease

### Imaging manifestations

The lesion locations were as follows: 7 cases, thoracic vertebrae; 3 cases, cervical vertebrae; 1 case, lumbar vertebrae; and 1 case, sacral vertebrae. Eleven patients had a single lesion, and one had multiple lesions (single lesion in spine and another single lesion in the manubrium). Vertebral body involvement was limited to a single level in 8 patients and was multi-level in 4 patients. Lesion diameters ranged from 2.9 to 7.3 cm, with the mean diameter being 4.9 cm (Table 1).

**Fig. 1:**
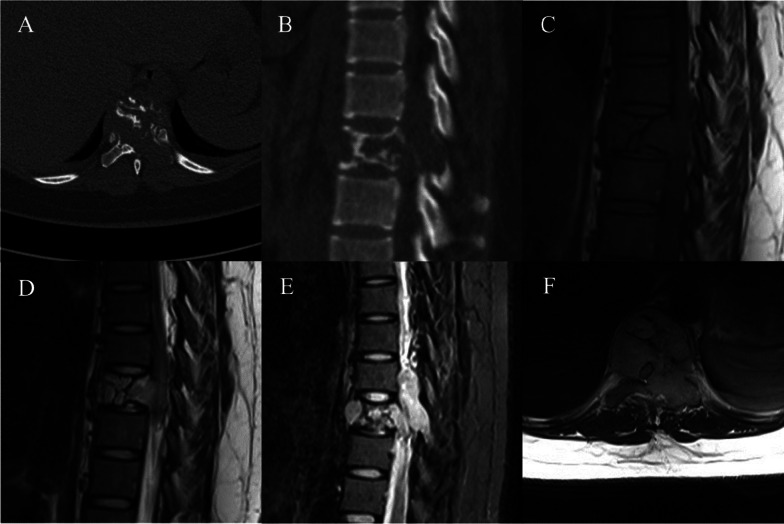
A 21-year-old woman with EHE (case 2). **a** Axial CT showing an ill-defined expansile osteolytic lesion of T9 with extension into the left posterior elements, containing thickened residual bone trabeculae and no sclerotic margin. **b** Sagittal CT showing a mild vertebral compression. **c** MR T1WI sagittal view showing the intermediate signal intensity (SI) of the lesion with a low-SI septum. **d** T2WI sagittal view showing that the tumor has high SI. **e** Fat suppression T2 sequence showing high SI of the lesion. **f** MR T2WI axial view showing a soft-tissue mass encroaching the spinal canal, which caused spinal cord compression

**Fig. 2:**
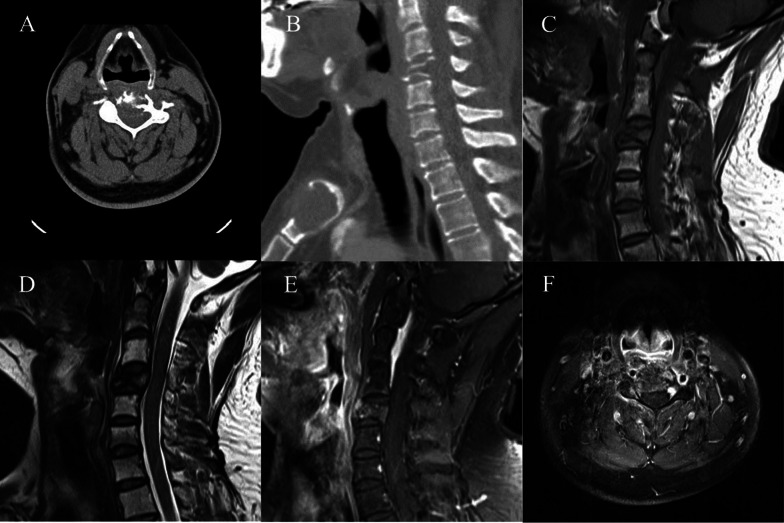
A 37-year-old man with EHE (case 4). **a** Axial CT showing an ill-defined expansile osteolytic lesion of C4 without a sclerotic margin, containing thickened residual bone trabeculae. **b** Sagittal CT showing multiple lesions of the manubrium and C4 vertebra. **c** MR T1WI sagittal view showing the intermediate SI of the lesion. **d** T2WI sagittal view showing that the tumor has slightly high SI. **e** Enhanced MR scan in the sagittal view showing heterogeneous enhancement of the mass. **f** Enhanced MR scan axial view showing that the mass had extended into the posterior elements

In nine cases, the lesions were centered in the vertebral body and extended into the posterior elements (Fig. 1), while in the remaining three cases, the lesions were centered in the posterior elements and extending into the vertebral body. Five cases showed low density on CT, 2 cases showed high density, and 5 cases showed isodense. Expansile osteolysis was noted in 10 cases. Eleven cases showed residual bone trabeculae. No sclerotic margin was observed in 11 cases. Six cases showed vertebral compression (Fig. 2). A surrounding soft-tissue mass was found in 11 cases, with the mass protruding into the spinal canal in six cases and surrounding the spinal canal in five cases (Fig. 1). MRI was performed for 11 patients, all of whom showed isointensity on T1WI (11/11), hyperintensity (9/11) or slight hyperintensity (2/11) on T2WI, and hyperintensity (11/11) on fat-suppressed T2WI (Fig. 1 C-F). In ten cases in which contrast-enhanced MR scanning was performed, a marked enhancement pattern was noted (Fig. 2 F). Detailed imaging manifestations of all cases are shown in Table 2.

**Table 2:** CT and MR manifestations of 12 spinal EHE patients

No	Density	Expansile osteolysis	Residual bone trabeculae	Sclerotic rim	Vertebral compression	Defined border	Majority of the lesion	Signal homogeneity	T1WI	T2WI	Fat suppression	Enhancement
1	Low	+	+	−	−	Ill	Vertebral body	Heterogeneous	Isointense	Hyperintense	Hyperintense	Obvious
2	Isodense	+	+	−	+	Ill	Vertebral body	Homogeneous	Isointense	Hyperintense	Hyperintense	−
3	Isodense	+	+	−	−	Well	Posterior elements	Heterogeneous	Isointense	Slightly hyperintense	Hyperintense	Obvious
4	High	+	+	−	+	Ill	Vertebral body	Homogeneous	Isointense	Hyperintense	Hyperintense	Obvious
5	Isodense	−	+	−	+	Well	Vertebral body	Homogeneous	Isointense	Hyperintense	Hyperintense	Obvious
6	Low	+	+	−	−	Well	Vertebral body	Homogeneous	Isointense	Hyperintense	Hyperintense	Obvious
7	Isodense	−	−	−	−	Well	Posterior elements	Homogeneous	Isointense	Hyperintense	Hyperintense	Obvious
8	Low	+	+	+	−	Ill	Vertebral body	Heterogeneous	Isointense	Hyperintense	Hyperintense	Obvious
9	High	+	+	−	+	Well	Vertebral body	Homogeneous	Isointense	Hyperintense	Hyperintense	Obvious
10	Isodense	+	+	−	+	Ill	Vertebral body	Homogeneous	Isointense	Hyperintense	Hyperintense	Obvious
11	Low	+	+	−	−	Well	Posterior elements	−	−	−	−	−
12	Low	+	+	−	+	Ill	Vertebral body	Heterogeneous	Isointense	Slightly hyperintense	Hyperintense	Obvious

## Discussion

EHE is a rare vascular tumor that originates from vascular endothelial or pre-endothelial cells, with an epithelioid and histiocytoid appearance. EHE can affect any soft tissue of the body, various parenchymatous organs, and bone [1]. EHE of the spine is more infrequent. According to the EHE cases included in the International Hemangioendothelioma, Epithelioid Hemangioendothelioma, and Related Vascular Disorders Support Group, the lesions often occur in a single organ (64%), with the liver accounting for the highest proportion (34%), followed by the lung (21%), the bone (19%), and others (26%) [15]. In the current 2013 World Health Organization (WHO 2013) Classification of Tumors of Soft Tissue and Bone, EHE was defined as lesions that fall into the category of locally aggressive tumors with metastatic potential [16]. Previous studies have indicated approximately 20–60% of cases were present with metastatic disease [17–19].

Histologically, EHE manifests as epithelioid endothelial cells arranged in nests or cords with infiltrative growth into surrounding tissues. The tumor cells can present with vascular differentiation, forming lumens of various size that occasionally contain erythrocytes, which are referred to as intracytoplasmic vacuoles. Immunohistochemically, these tumors appear to express endothelial markers such as EGR, CD31, CD34, F VIII-Rag, which are usually expressed by EHE as well as other types of vascular or soft-tissue tumors, indicating that these markers have poor specificity [2, 3, 20]. Epithelioid markers can occasionally be expressed by EHE [1]. The FLI-1 protein has been shown to show certain effectiveness in the identification of vascular tumors, including EHE, and it was also recognized as an endothelial cell marker with a combined sensitivity and specificity superior to those of widely used endothelial markers alone [21, 22]. In terms of molecular genetics, according to literature reports, t (1;3) (p36.23; q25.1) is a highly specific chromosomal translocation for EHE, resulting in a fusion between the WW domain‐containing transcription regulator 1 (WWTR1) gene on 3q25 and the calmodulin‐binding transcription activator 1 (CAMTA1) gene on 1p36, which is present in nearly 90% of EHE cases [23–25].

Spinal EHE lacks specific clinical symptoms, and patients usually present with focal neck or back pain, which may be accompanied by weakness, numbness, or paresthesia of extremities. These manifestations mainly depend on the location and size of the lesion. In our cases, most of the patients presented with typical symptoms such as neck or back pain, but the chief complaint of one patient was solely chest pain.

Treatment options for spinal EHE include preoperative embolization, surgical resection, radiotherapy, and chemotherapy. Treatment planning must be based on the diagnosis of spinal EHE by biopsy and extent on imaging, after which the specific plan should be formulated according to the patient's medical history and clinical symptoms, but there is still no unified treatment standard at present [5]. Because EHE presents with low-grade malignancy with the potential of metastasis, therapeutic management should be relatively aggressive. When conditions permit, surgery is preferred for most cases, which can be combined with radiotherapy and/or chemotherapy. Spinal EHE has low recurrence rate and long-term survival outcome after definitive surgery [5, 11]. The efficacy of preoperative embolization, wide resection, and radiotherapy for spinal EHE has been reported in the literature [8, 11, 13] but the evidence for chemotherapy is still inconclusive [9, 14].

Previous studies have suggested that spinal EHE lacks specific imaging manifestations [5, 12]. In our study, we found that some features appeared frequently, which may provide some valuable information for the imaging diagnosis. In general, EHE often occurs in the thoracic vertebra and manifests as mild expansile osteolysis, with ill-defined boundaries and surrounding soft-tissue mass but an uncommon sclerotic rim. It is prone to pathological compression fractures, which appear as a high signal on T2WI. EHE is a low-grade malignant vascular neoplasm, a typical EHE is characterized by a hyperintense signal on T2WI and marked enhancement on contrast-enhanced scanning due to the vascular elements of the tumor. In our study, we found that all cases showed isointensity on T1WI, hyperintensity or slight hyperintensity on T2WI, obvious enhancement. These MRI manifestations have also been reported in previous case reports[7, 9]. The low-grade malignancy biological behavior of EHE results in a propensity to present with an ill-defined border, surrounding soft-tissue mass, no sclerotic rim, pathological compression fracture, and other signs. Nonetheless, EHE remains difficult to diagnose, some of the imaging features lack specificity, overlapping with other tumors. The histopathology will be helpful for diagnosis.

For diagnosis of EHE, CT and MRI have their advantages and disadvantages. MRI has superior soft tissue contrast and has proved to be especially advantageous in identifying vascular tissue [26], therefore can be useful in revealing the pathologic characteristics of the EHE. CT is more valuable in detecting the change of bone substance than MRI, such as residual bone trabeculae and sclerotic rim.

EHE needs to be differentiated from hemangioma, which is the most common vascular tumor of the spine. A symptomatic hemangioma can also present as mild expansile osteolysis and hyperintensity on T2WI. A soft-tissue mass can occasionally be delineated and encroachment of the spinal canal may occur [27]. We found that in comparison with hemangioma, EHE appears to show more obvious expansile osteolysis with an ill-circumscribed boundary, more sparse and coarse residual bone trabeculae, and more common soft-tissue masses (Fig. 1) and vertebral compression (Fig. 2). These features may be explained by the fact that the biological behavior of an EHE is more active and more malignant than that of hemangioma. In addition, symptomatic hemangioma is mostly a single-center lesion, whereas EHE can occur in multiple sites[14]. If multiple vascular lesions are found, EHE should be suspected (Fig. 2 B).

This work represents a preliminary analysis of spinal EHE imaging features. Although some valuable findings were obtained, the study had several limitations. First, our study included a limited number of EHE cases. Second, the integrity and homogeneity of data cannot be guaranteed since the imaging examinations of some patients were not comprehensive, which resulted from the limited statistical power for this retrospective study. In the future, we will further expand the sample size in a prospective design to validate and refine the results obtained in this study. Furthermore, we will explore the diagnostic value of advanced imaging examinations.

## Conclusions

In summary, EHE often occurs in the thoracic vertebra and may occur at multiple sites. It usually manifests as mild expansile osteolysis with ill-defined boundaries and surrounding soft-tissue mass. Residual bone trabeculae are frequently found in the destruction area, but a sclerotic rim is uncommon. A pathological compression fracture can develop in these cases. MR always shows characteristic signal changes of the vascular tumor, showing isointensity on T1WI, hyperintensity on T2WI, and marked enhancement on contrast-enhanced scanning.

## Data Availability

The datasets used and/or analyzed during the current study are available from the corresponding author on reasonable request.

## Abbreviations

C: Cervical
EHE: Epithelioid hemangioendothelioma
F: Female
L: Lumbar
M: Male
S: Sacral
SI: Signal intensity
T: Thoracic

## References

[1] Sardaro A, Bardoscia L, Petruzzelli MF, Portaluri M (2014). Epithelioid hemangioendothelioma: an overview and update on a rare vascular tumor. Oncol Rev.

[2] Mentzel T, Beham A, Calonje E, Katenkamp D, Fletcher CD (1997). Epithelioid hemangioendothelioma of skin and soft tissues: clinicopathologic and immunohistochemical study of 30 cases. Am J Surg Pathol.

[3] Flucke U, Vogels RJ, de Saint Aubain Somerhausen N (2014). Epithelioid Hemangioendothelioma: clinicopathologic, immunhistochemical, and molecular genetic analysis of 39 cases. Diagn Pathol.

[4] Rosenberg A, Agulnik M (2018). Epithelioid hemangioendothelioma: update on diagnosis and treatment. Curr Treat Options Oncol.

[5] Albakr A, Schell M, Drew B, Cenic A (2017). Epithelioid hemangioendothelioma of the spine: case report and review of the literature. J Spine Surg.

[6] Kelahan LC, Sandhu FA, Sayah A (2015). Multifocal hemangioendothelioma of the lumbar spine and response to surgical resection and radiation. Spine J.

[7] Chen PK, Lin QT, Feng YZ, Weng ZP, Cai XR (2020). Epithelioid hemangioendothelioma of spine: a case report with review of literatures. Radiol Case Rep.

[8] Sybert DR, Steffee AD, Keppler L, Biscup RS, Enker P (1995). Seven-year follow-up of vertebral excision and reconstruction for malignant hemangioendothelioma of bone. Spine (Phila Pa 1976).

[9] Ellis TS, Schwartz A, Starr JK, Riedel CJ (1996). Epithelioid hemangioendothelioma of the lumbar vertebral column: case report and review of literature. Neurosurgery.

[10] Larochelle O, Périgny M, Lagacé R, Dion N, Giguère C (2006). Best cases from the AFIP: epithelioid hemangioendothelioma of bone. Radiographics.

[11] Luzzati A, Gagliano F, Perrucchini G, Scotto G, Zoccali C (2015). Epithelioid hemangioendothelioma of the spine: results at seven years of average follow-up in a series of 10 cases surgically treated and a review of literature. Eur Spine J.

[12] Ma J, Wang L, Mo W, Yang X, Xiao J (2011). Epithelioid hemangioendotheliomas of the spine: clinical characters with middle and long-term follow-up under surgical treatments. Eur Spine J.

[13] Aflatoon K, Staals E, Bertoni F (2004). Hemangioendothelioma of the spine. Clin Orthop Relat Res.

[14] Boutin RD, Spaeth HJ, Mangalik A, Sell JJ (1996). Epithelioid hemangioendothelioma of bone. Skeletal Radiol.

[15] Lau K, Massad M, Pollak C (2011). Clinical patterns and outcome in epithelioid hemangioendothelioma with or without pulmonary involvement: insights from an internet registry in the study of a rare cancer. Chest.

[16] Fletcher CDHP, Mertens F (2013). 2013WHO classification of tumours of soft tissue and bone WHO classification of tumours.

[17] Guo Q, Xue J, Xu L, Shi Z, Zhou B (2017). The clinical features of epithelioid hemangioendothelioma in a Han Chinese population: a retrospective analysis. Medicine (Baltimore).

[18] Stacchiotti S, Miah AB, Frezza AM (2021). Epithelioid hemangioendothelioma, an ultra-rare cancer: a consensus paper from the community of experts. ESMO Open.

[19] Shiba S, Imaoka H, Shioji K (2018). Clinical characteristics of Japanese patients with epithelioid hemangioendothelioma: a multicenter retrospective study. BMC Cancer.

[20] Makhlouf HR, Ishak KG, Goodman ZD (1999). Epithelioid hemangioendothelioma of the liver: a clinicopathologic study of 137 cases. Cancer.

[21] Rossi S, Orvieto E, Furlanetto A, Laurino L, Ninfo V, Dei Tos AP (2004). Utility of the immunohistochemical detection of FLI-1 expression in round cell and vascular neoplasm using a monoclonal antibody. Mod Pathol.

[22] Folpe AL, Chand EM, Goldblum JR, Weiss SW (2001). Expression of Fli-1, a nuclear transcription factor, distinguishes vascular neoplasms from potential mimics. Am J Surg Pathol.

[23] Errani C, Zhang L, Sung YS (2011). A novel WWTR1-CAMTA1 gene fusion is a consistent abnormality in epithelioid hemangioendothelioma of different anatomic sites. Genes Chromosom Cancer.

[24] Tanas MR, Sboner A, Oliveira AM (2011). Identification of a disease-defining gene fusion in epithelioid hemangioendothelioma. Sci Transl Med.

[25] Shibuya R, Matsuyama A, Shiba E, Harada H, Yabuki K, Hisaoka M (2015). CAMTA1 is a useful immunohistochemical marker for diagnosing epithelioid haemangioendothelioma. Histopathology.

[26] Woertler K (2003). Benign bone tumors and tumor-like lesions: value of cross-sectional imaging. Eur Radiol.

[27] Erlemann R (2006). Imaging and differential diagnosis of primary bone tumors and tumor-like lesions of the spine. Eur J Radiol.

